# Impact of COVID-19 Pandemic on Healthcare Utilization among Patients with Type 2 Diabetes Mellitus: A Systematic Review

**DOI:** 10.3390/ijerph20054577

**Published:** 2023-03-04

**Authors:** Norizzati Amsah, Zaleha Md Isa, Norfazilah Ahmad, Mohd Rizal Abdul Manaf

**Affiliations:** Department of Community Health, Faculty of Medicine, Universiti Kebangsaan Malaysia, Jalan Yaacob Latif, Bandar Tun Razak, Cheras, Kuala Lumpur 56000, Malaysia

**Keywords:** impact, COVID-19 pandemic, diabetes mellitus, healthcare utilization

## Abstract

As the COVID-19 pandemic continues, healthcare services have been grossly overwhelmed by the pandemic. Due to this circumstance, routine care for individuals with type 2 diabetes mellitus (T2DM) has been temporarily disrupted. The purpose of this systematic review was to summarize the evidence regarding the impact of the COVID-19 pandemic on healthcare utilization among patients with T2DM. A systematic search was conducted in the Web of Science, Scopus, and PubMed databases. The process of identifying the final articles followed the PRISMA guidelines. The inclusion criteria were articles published between 2020 and 2022, written in English, and studies focusing on the research question. Any proceedings and books were excluded. A total of fourteen articles relevant to the research question were extracted. Following that, the included articles were critically appraised using the Mixed Method Appraisal Tool (MMAT) and the Joanna Briggs Institute Critical Appraisal Tool to assess the quality of the studies. The findings were further classified into three themes: reduced healthcare utilization among T2DM patients in routine healthcare services, a surge of telemedicine usage, and delay in the delivery of healthcare services. The key messages include demands for monitoring the long-term effects of the missed care and that better preparedness is crucial for any pandemic in the future. A tight diagnostic workup at the community level and regular follow-ups are crucial in managing the impact of the pandemic among T2DM patients. Telemedicine should be on the agenda of the health system to maintain and complement healthcare services. Future research is warranted to determine effective strategies to deal with the impact of the pandemic on healthcare utilization and delivery among T2DM patients. A clear policy is essential and should be established.

## 1. Introduction

The World Health Organization (WHO) declared the coronavirus disease 2019 (COVID-19) pandemic on 11 March 2020. The pandemic led to unprecedented changes in the utilization of health care services due to restriction orders and lockdowns [1]. Gellman and Turner defined healthcare utilization as the use of the healthcare system by individuals for the purpose of preventing and treating health problems, promoting health and well-being, or getting information about one’s health condition and prognosis [2]. According to Saeed et al. (2012), the term “healthcare utilization” refers to the extent to which an individual interacts with any recognized medical facility or health care provider [3]. Access to health care services was influenced by contextual characteristics, individual characteristics, health behaviors, and outcomes that can determine an individual’s health status and consumer satisfaction [4,5].

With significant resources devoted to the treatment of patients with COVID-19, the reorganization of human resources and those working in outpatient care witnessed the prioritization of the health system in combating COVID-19, while treatments for people with chronic diseases were partially or completely disrupted [6,7]. Many human resources and staff were diverted from their normal activities to provide treatment and management plans for COVID-19 cases [8]. Furthermore, the pandemic had a significant impact on healthcare utilization, forcing many chronic patients, such as those with hypertension and stroke, to reschedule their follow-up visits, and some even missed their routine check-ups [9]. According to Fekadu et al., which showed that delivering routine comprehensive care for chronic patients such as non-communicable disease patients was disrupted due to closures of healthcare facilities, unavailability of public transportation, or reductions in services [10].

The first case in Malaysia was reported on 25 January 2020, involving three Chinese nationals who had close contact with infected people in Singapore [11]. The Malaysian government declared a state of emergency on 12 January 2021, in order to push for a restriction control order and as a preventive measure to better prepare for the critical nationwide disaster [12]. The Movement Control Order (MCO) has been enacted by the government to prevent the virus from spreading and has had an influence on the general health of the population [13]. In addition, a study in Malaysia showed most respondents (78%) only left the house once or twice a week due to fear of the virus and the restriction order [14]. All the COVID-19 patients were sent to public hospitals for treatment and isolation. The rise in COVID-19 cases has overwhelmed the public healthcare system and overloaded healthcare resources, and this situation indirectly affects healthcare utilization across all categories of healthcare services [15].

Type 2 diabetes mellitus (T2DM) continues to constitute a crucial public health issue globally that requires ongoing healthcare management. T2DM is a medical disorder that has a significant impact on affected individuals and society due to the high expenditures involved with its care and complications [16,17,18,19]. The situation worsens during the COVID-19 pandemic because T2DM increases the risk of hospitalization and has a higher risk of incident mortality due to severe infection from COVID-19 compared to those without T2DM [20,21]. A study in the United States demonstrated that there was a significant surge of more than 30% in mortality during the pandemic due to diabetes-related deaths [22]. Many studies discuss the prevention of diabetes complications and emphasize the importance of regular follow-up to achieve good diabetes control [23].

Based on recent findings, T2DM patients were most impacted by the reduction in healthcare resources due to COVID-19 [24]. Patients with T2DM were unable to access medical care and faced multiple challenges towards diabetes self-care [25,26]. Furthermore, many pressing problems of T2DM patients would not be adequately addressed during this crisis due to reduced service capacity. Nevertheless, to date, the literature on the impact of COVID-19 on the healthcare utilization of T2DM patients is limited. The existing studies mainly focused on the impact of COVID-19 on health outcomes and health status [27,28], disease progression [29], and disease management [25,30,31], instead of healthcare usage among T2DM patients. For example, a study in our local setting showed that the implementation of MCO had a slight impact on diabetic control among T2DM patients in the study population [32]. Previous studies were more focused on primary studies, and there are limited studies in terms of systematic reviews that have been discussed on the impact of COVID-19 on healthcare utilization among T2DM patients. Therefore, the purpose of this systematic review was to summarize the evidence regarding the impact of the COVID-19 pandemic on healthcare utilization among patients with T2DM.

## 2. Materials and Methods

This systematic review followed the recommendations of the Preferred Reporting Items for Systematic Reviews and Meta-Analyses (PRISMA) [33]. The protocol (CRD42022374035) was registered with the International Prospective Register of Systematic Reviews (PROSPERO). The objective of this review was to summarize the evidence regarding the impact of the COVID-19 pandemic on healthcare utilization among patients with T2DM.

### 2.1. Formulation of the Research Question

In this review, the formulation of the research question was based on the PEO (population, exposure, outcome) mnemonic concept [34]. The components of the PEO were established as follows: (1) Population: type 2 diabetes mellitus; (2) Exposure: COVID-19 pandemic; and (3) Outcome: healthcare utilization. The PEO concept guided the formulation of the main research question, “What is the impact of the COVID-19 pandemic on the healthcare utilization among T2DM patients?”

### 2.2. Searching Strategies

**Figure 1:**
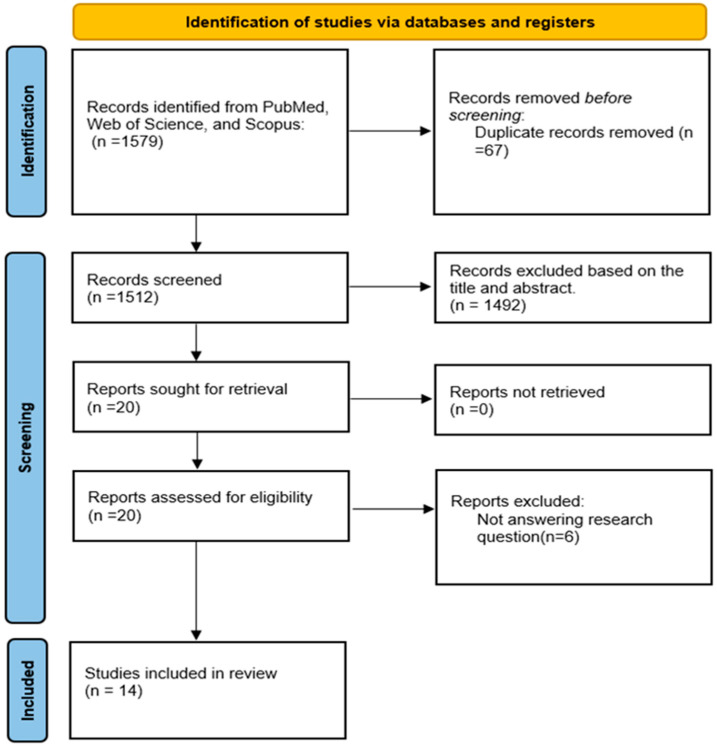
PRISMA flow diagram.

The literature search was conducted in November 2022, using the Web of Science (WoS), Scopus, and PubMed databases. The systematic search strategies were based on the PRISMA flow, which consists of the identification, screening, and eligibility stages (Figure 1).

### 2.3. Identification

Relevant keywords using the Medical Subject Headings (MeSH) terms were identified during this stage. Specific search strings were developed using Boolean operators and identified keywords. The search string and the systematic search in electronic databases from PubMed, WoS, and Scopus on the keywords were employed in the identification procedure as indicated in Table 1, which resulted in the retrieval of 1579 records. In addition to the above-mentioned databases, the search was carried out using the snowballing technique, which involved looking for references from the first search to avoid missing any related articles. The database records were extracted and organized in an Excel spreadsheet for screening. Any duplication of articles was deleted.

### 2.4. Screening Using Inclusion and Exclusion Criteria

At this stage, four authors have independently assessed each article’s title and abstract to determine whether it meets the review’s inclusion and exclusion criteria. The inclusion criteria for article selection were: (1) the articles were published between 2020 and 2022; (2) they were written in English; and (3) the articles were specifically relevant to the research question. For the exclusion criteria, any conference proceedings, book chapters, editorial letters, and reports were excluded. Non-relevant articles that were not related to our research questions and did not fulfill our inclusion criteria were excluded at this stage.

### 2.5. Eligibility

A total of 20 full-text articles were successfully retrieved for eligibility. The potential articles identified during the main screening were kept, and the full text was independently reviewed by the two reviewers in detail according to the research question. Any non-related articles were removed. Any disagreement that arose between each pair of reviewers was determined by the third reviewer. The remaining fourteen articles were reviewed using the quality appraisal tool.

### 2.6. Quality Assessment

Quality appraisal was conducted using the Mixed Method Appraisal Tool (MMAT) [35]. The MMAT evaluates the quality of qualitative, quantitative, and mixed-method studies. It focuses on methodological criteria and includes five core quality criteria for each of the following five categories of study designs: (1) quantitative, (2) qualitative, (3) randomized controlled, (4) non-randomized, and (5) mixed methods [36]. The marks of MMAT for this review were 80–100%, as presented in the Supplementary Materials, which indicates that the included articles have a good quality appraisal with clear study objectives and an appropriate study design. For quality assessment of the systematic review, the Joanna Briggs Institute Critical Appraisal Tool was used.

### 2.7. Data Abstraction and Analysis

Four authors independently collected data from the selected articles, including the authors’ names, years, countries, study designs, and findings. A matrix table was created using the data extracted from each study (Table 2). During data analysis, all information will undergo thematic synthesis, which has three stages: coding, development of descriptive themes, and generation of analytical themes [37]. At this point, data analysis was carried out using a method in which the authors coded the key findings of the included studies until specific themes were developed. The emerging themes revealed certain patterns, relations, and explanations of the combined data. The authors looked for similarities and differences in the matrix table to generate results and themes. Information that had a similarity was categorized as having one theme, and this process was repeated to obtain valid conclusions.

### 2.8. Data Analysis

The study designs and reported outcomes varied significantly; therefore, a meta-analysis could not be conducted on all included studies. Studies were excluded from the meta-analysis if the reviewers considered them to be insufficient to contribute meaningfully to the body of evidence. The pool estimates for the surge in telemedicine usage among T2DM and its 95% confidence intervals (CIs) were calculated, and analyses were conducted using the statistical package ‘dosresmeta’ in R statistical software version 4.2.1 (Robert Gentleman and Ross Ihaka from the Statistic Department of the University of Auckland, Auckland, New Zealand), while I^2^ statistics was used to test the heterogeneity of the studies.

## 3. Results

The search yielded 20 articles from Web of Science, 921 articles from Scopus, and 638 articles from PubMed, resulting in 1579 unique hits. From the 1579 articles, 67 were duplicates, 1492 were excluded based on abstract screening, and 6 were excluded based on full-text screening. Only fourteen articles were included in the full-text assessment after rigorous selection screening, as shown in the PRISMA flow diagram in Figure 1. The findings from fourteen studies were included in this review, as shown in Table 2. Two eligible articles each were from the United Kingdom and the United States. One global study included surveys from 27 European countries and one each from Finland, Muscat, Singapore, Spain, and Japan. In terms of study design, four articles were cross-sectional studies, six articles were cohort studies, three were systematic reviews, and one was a scoping review.

Based on the pattern of the findings identified from the fourteen reviewed articles, similar and related data were grouped, and three main themes were derived. As presented in Table 3, the three themes are (i) reduced healthcare utilization among T2DM patients in routine healthcare services, (ii) surge in telemedicine usage, and (iii) delay in the delivery of healthcare services. A summary of reasons for reduced healthcare utilization in routine healthcare services is presented in Table 4.

### Meta-Analysis

**Figure 2:**
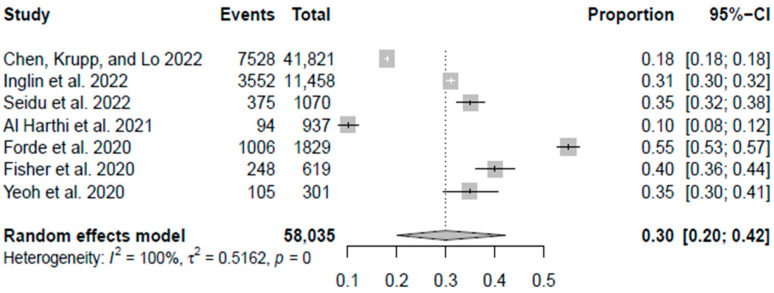
A forest plot of meta-analysis shows the surge in telemedicine usage among T2DM patients during the COVID-19 pandemic. (Chen, Krupp, and Lo 2022 [38], Inglin et al., 2022 [39], Seidu et al., 2022 [41], Al Harthi et al., 2021 [43], Forde et al., 2020 [49], Fisher et al., 2020 [50], and Yeoh et al., 2020 [51].

Due to data limitations, a meta-analysis on reduced healthcare utilization in routine healthcare services and the delay in the delivery of healthcare services could not be performed. The meta-analysis was only done on the surge in telemedicine usage. Out of 14 studies, seven have enough data to conduct a meta-analysis on the surge in telemedicine usage. The R program version 4.2.1 was used to conduct the analysis. A random effect model was used to calculate the combined increase in telemedicine usage. The pooled increase in telemedicine usage was 30%, with a 95% CI [20,21,22,23,24,25,26,27,28,29,30,31,32,33,34,35,36,37,38,39,40,41,42] as shown in the forest plot in Figure 2. The heterogeneity was assessed by the I^2^ statistics and was considered high heterogeneity at 100%. 

## 4. Discussion

As the COVID-19 pandemic spread around the world in the first quarter of 2020, most countries saw a decrease in healthcare services for non-COVID-related diseases like diabetes mellitus [52]. In this review, we discussed how the COVID-19 pandemic influenced healthcare utilization among T2DM patients. The findings of our review are further discussed in a few sections below.

### 4.1. Reduced Healthcare Utilization in Routine Healthcare Services

Based on our findings, the four included studies found consistent evidence of significant reductions in healthcare utilization among T2DM patients during the pandemic. Our findings showed that specific routine health check-ups among T2DM patients revealed a decrease in the proportion of patients who obtained HbA1c testing as well as a decrease in the overall utilization of non-emergent outpatient visits [38]. All gender and age groups experienced a reduction in HbA1c testing that was about half compared to the previous year, and the older patients were the most affected group [42]. This situation is consistent with a survey, which found that HbA1c values significantly worsened among older patients as a result of the reduction in HbA1c testing during the pandemic [53]. This condition may have been attributed to a reduction in the level of physical activity and increased rates of sedentary behavior during the COVID-19 pandemic [54].

In the United Kingdom, Carr et al. found the percentage of patients doing health check-ups, blood pressure monitoring, and body mass index monitoring was reduced by 76–88%, which was more prominent among older people and low-income families [44]. Similar to the study by Inglin et al., primary health care service usage among T2DM patients was significantly lower in 2020 compared to 2019, and the mean number of all contacts (appointments and remote consultations) per person decreased by 9.2% [39]. In the setting of the emergency department, a study in Turkey found that the number of emergency department visits during the pandemic was reduced by half when compared to the previous year [55]. Despite a significant decrease in emergency visit rate during the early phase of the lockdown period, a rebound effect was observed, as the number of emergency visits in 2020 exceeded the numbers of the previous year [39]. Nevertheless, the usage has gradually increased since the outbreak, but it has yet to return to normal [56]. Based on our findings, there are a few reasons for reduced healthcare utilization in routine healthcare services, as shown in Table 4. For example, the COVID-19 pandemic, lockdown, and restrictive measures affect accessibility and the organization of services drastically. Other than that, restrictions on outdoor physical activity, social isolation, and the reduction of clinical services are among the reasons for reduced healthcare utilization.

In comparison, Chen, Krupp, and Lo (2022) discussed their findings in hospital settings, including emergent and non-emergent settings, whereas Carr et al. focused on primary care settings [38,39,42,44].

### 4.2. Surge of Telemedicine Usage

In our review, nine studies discussed the increased use of telemedicine during the pandemic [38,39,41,43,46,48,49,50,51]. In the midst of the COVID-19 pandemic, the implementation of telemedicine has been promoted and accelerated [57]. Based on data from included studies through meta-analysis, we discovered a 30% surge in telemedicine usage. Chen, Krupp, and Lo found in their study that the percentage of patients with diabetes-related telehealth visits had increased by 18% in the United States [38]. Some countries introduced virtual clinics to provide diabetic care due to preventative measures such as lockdowns, cancellation of in-person appointments, and patients’ fear of becoming infected while attending clinics [58]. We discovered that the proportion of remote consultations in Finland was similar in both pre-lockdown years (56.3–59.5%) but increased to 88.0% during the lockdown in 2020, and Inglin et al. showed that three-quarters of diabetes-related health contacts were conducted remotely in 2020 [39].

Furthermore, a global survey involving 27 countries showed a significant increase in virtual contact with people with diabetes mellitus via telephone, email, and video consultations [30]. In a survey of healthcare professionals (HCPs) from 47 countries, HCPs highlighted the usage of telemedicine, which included online video consultations via Zoom, Skype, WhatsApp, and Facebook Messenger [24]. One-third of them use face-to-face and phone consultations to provide routine chronic disease management care for their patients, and 45% stated that all of the appointments were shifted to remote consultations by phone [24]. A similar finding by Yeoh et al. found that the majority of T2DM patients were willing to explore tele-consultation options, and the majority of them were able to reach their doctor through either phone, messaging, or email despite not attending clinic [51].

In Singapore, telephone consultations (92%) were the most common, and 35% used video consultations [41]. In terms of virtual consultations, 36% reported that the consultation time was the same or slightly longer than before the pandemic. In terms of those who were previously difficult to engage, 39% believed that a larger range of communication approaches had a moderately good effect, whereas 20.9% reported no benefit [41]. During an emergency, the teleconsultations approach may have helped people obtain basic diabetes follow-up advice without being exposed to the risk of infection by visiting a hospital [59]. In addition, telephone consultations and messenger services were used to maintain communication with patients [60]. Participants were able to get dietary and lifestyle guidance, adherence reinforcement, and therapy modifications as needed [61].

According to Tourkmani et al., newly implemented telemedicine care in Saudi Arabia had a considerable positive effect on glycaemic control among T2DM [58]. Similar to a study in Australia, patients with T2DM who obtained care via telehealth consultations during the COVID-19 lockdown had better glycaemic control, and the admission rates were not higher than in the pre-COVID-19 period [62]. Under these conditions, telehealth consultation provides an important care delivery option for diabetic patients. The healthcare professionals believed that video consultations provided effective screening for consultations, avoiding crowds and waiting lists, allowing for fast resolutions of simple, minor disorders, and reducing workloads and costs in healthcare facilities [63].

Nevertheless, Fisher et al. found that 40% reported a move to telemedicine, and nearly half expressed reduced overall satisfaction with these visits compared to the pre-pandemic setting [50]. Furthermore, there were limitations to telemedicine among older groups, as they were unwilling to explore teleconsultation and were more comfortable with the traditional method [51]. Despite the effectiveness of telemedicine programs, there are still certain challenges, such as devices and internet availability [64]. Another example is that some insulin pump patients and caregivers had difficulty downloading and sharing pump details with their physicians, as well as modifying pump settings [65].

Comparatively, six of the seven included studies were conducted at the national level, whereas Forde et al. (2020) conducted a global survey that involved 27 countries with varying populations, healthcare systems, and resources [49]. Despite differences in location and study design, the included studies revealed a surge in telemedicine usage.

### 4.3. Delay in the Delivery of Healthcare Services

In our review, four studies showed a pattern of delay in the delivery of healthcare services [40,45,47,50]. A study by Forde et al. found a significant drop in the level of diabetes care delivered during the pandemic across Europe [49]. In the United States, it was found that approximately 40% of respondents indicated that all their diabetic healthcare appointments were cancelled or postponed at the time. Self-management support, diabetes education, and psychological support were the most affected areas, as stated by 21%, 63%, and 34% of respondents, respectively [50]. Medical care delivery has been significantly interrupted during the COVID-19 pandemic, resulting in medical treatment delays [66]. Preventive measures for the spread of COVID-19 have led to delays in in-person health care services [67]. According to Mohseni et al., the challenges of routine chronic disease care during the COVID-19 pandemic were transportation issues, limited self-care practice, unaffordable medicine, shortages of staff and medication, limited inpatient capacity, delayed care seeking, and lockdowns of standard outpatient clinics [45].

In our review, we found that there was a significant increase in delayed clinic visits among women compared to men [40]. Female gender, higher levels of education, more concerns about the pandemic, and poorer self-rated physical health were associated with delayed medical care [66]. Other than that, one-third of older individuals said their medical care has been delayed since the outbreak began and has negatively affected their health [66]. In some outpatient services, after the start of lockdown measures, appointments for non-COVID-19-related illnesses were not available or were restricted to emergency cases only [68].

These findings were inconsistent with a study in Muscat, which showed the majority of patients who had attended a diabetes clinic prior to the pandemic continued to receive diabetes care [43]. Despite the halting of standard services and the implementation of new ways of consultation, such as phone consultation, access to basic diabetes treatment was maintained [43]. The same situation can be seen in Singapore during the pandemic, as the majority of patients with diabetes were able to access health care and diabetes medical supplies, demonstrating their effective strategies in handling the pandemic [51].

Based on our findings, Maeda et al. (2022) utilized information from insurance claim data from the Joint Health Insurance Society [40], while Fisher et al. (2020) utilized data from the Taking Control of Your Diabetes (TCOYD) research registry [50].

### 4.4. Strength and Limitation

Our review has several strengths. We synthesized the most recent data reported on primary studies up to the end of December 2022 and utilized the PRISMA guidelines. There were several limitations to this review and the included studies. There were limited keywords used in our studies; however, we minimized the biases through the snowballing technique, which involved looking for references from the first search to avoid missing any related articles. Some studies may have focused on similar themes that were removed during the screening procedure due to the use of different keywords and titles. We did not use databases such as ProQuest because we do not have direct access to them. In our review, we utilized at least three databases, namely Web of Science (WoS), Scopus, and PubMed. Language bias should be considered, as we only included articles published in English. Other than that, for the meta-analysis, we did not proceed with subgroup analysis and publication biases as there are limited articles and data. Despite these limitations, this systematic review synthesizes recent evidence regarding the impact of the COVID-19 pandemic on healthcare utilization among T2DM patients, which may serve as a guide to improve healthcare service delivery strategies in any future pandemic.

## 5. Conclusions

The COVID-19 pandemic had an impact on healthcare utilization, usage of telemedicine, and healthcare delivery among T2DM patients. The key messages include demands for monitoring the long-term effects of the missed care and that better preparedness is crucial for any pandemic in the future. A tight diagnostic workup at the community level and regular follow-ups are crucial in managing the impact of the pandemic among T2DM patients. Telemedicine should be on the agenda of the health system to maintain and complement healthcare services. Future research is warranted to determine effective strategies to deal with the impact of the pandemic on healthcare utilization and delivery among T2DM patients. A clear policy is essential and should be established.

## Figures and Tables

**Table 1:** Keyword search in the identification process.

Database	Search String
Web of Science	(“effect” OR “impact” OR “implication”) AND (“COVID-19 pandemic” OR “pandemic”) AND (“diabetes*” OR “diabetes mellitus”) AND (“healthcare utilization” OR “healthcare utilize*” OR “healthcare usage”)
Scopus	
PubMed	

**Table 2:** Findings from the included studies.

	Author, Year	Location	Study Design	Study Setting	Findings
1.	Chen, Krupp, and Lo 2022 [38]	United States	Cohort	Outpatient visits (in-person and telehealth) Emergency department visits and inpatient admissions.	There were decreases in the proportion of patients who obtained HbA1c testing. There were decreases in both the proportion of patients with diabetes-related in-person office visits and the number of visits per patient. Overall utilization of non-emergent outpatient visits declined. The proportion of patients with diabetes-related telehealth visits increased by 18%.
2.	Inglin et al., 2022 [39]	Finland	Retrospective cohort study	All primary healthcare and specialized healthcare.	During the lockdown period, the number of diabetes-related contacts decreased significantly but quickly increased again to nearly the same level as in 2019. Overall, healthcare usage was lower in the pandemic year, with proportionally 9% fewer contacts per person and a proportionally 9% lower proportion of patients making any contact. The proportion of remote consultations was similar in both years in the pre-lockdown period (56.3–59.5%), but then increased to 88% during the 2020 lockdown.
3.	Maeda et al., 2022 [40]	Japan	Retrospective cohort study	Insurance claims data from the Joint Health Insurance Society	There was a significant increase in delayed clinic visits during the pandemic, and women had significantly fewer clinic visits during the COVID-19 pandemic than men.
4.	Seidu et al., 2022 [41]	United Kingdom	Cross-sectional study	Primary care services	The most common consultation methods used to provide diabetes care during the pandemic were telephone consultation (92.0%), face-to-face consultation (80.2%), and video consultation (35%).
5.	Palanca et al., 2021 [42]	Spain	Cross-sectional study	Hospital, primary care centres within the city of Valencia, peripheral primary care centers away from the metropolitan area, and nursing homes.	During full lockdown, about 50% of participants experienced a reduction in HbA1c testing, and the oldest participants were the most affected group.
6.	Al Harthi et al., 2021 [43]	Muscat	Retrospective Cohort study	Primary care setting	Most patients received face-to-face consultation alone (57.4%), followed by combined face-to-face and telephone consultation (32.4%), and telephone consultation alone (10%). Most patients continued to receive diabetes care following the pandemic announcement by taking initiatives through phone consultation.
7.	Carr et al., 2021 [44]	United Kingdom	Retrospective cohort	Primary care setting	In primary care, the rate of performing health checks was reduced by 76–88%, commonly among older people and low-income families.
8.	Mohseni et al., 2021 [45]	-	Systematic review	Primary care setting and secondary care setting	Outpatient and secondary care facilities have been pushed to limit or cancel their routine health service provision to mobilize healthcare providers to other high-pressure areas. Access to inpatient care is diminished for patients with other conditions.
9.	Yin et al., 2021 [46]	-	Systematic review	Inpatient and outpatient services	The COVID-19 pandemic has led to increased use of telemedicine.
10.	Sciberras et al., 2020 [47]	-	Systematic review	Outpatient services.	Most outpatient services were temporarily halted during the pandemic, while those that continued their services were challenged due to staff reduction.
11.	Wicaksana et al., 2020 [48]	-	Scoping review	Inpatient and outpatient services	Emphasized the use of telehealth consultation for blood sugar monitoring, and telemedicine using mobile phones is useful for delivering diabetes education.
12.	Forde et al., 2020 [49]	27 European countries: Belgium, Bosnia and Herzegovina, Croatia, Cyprus Czechia, Denmark, Estonia, Finland, France, Germany, Greece, Ireland, Italy, Latvia, Malta, Netherlands, Norway, Poland, Portugal, Romania, Spain, Sweden, Switzerland, Turkey, Ukraine, United Kingdom (UK)	Cross-sectional	27 countries with varying populations, healthcare systems, and resources.	Large increase in virtual contact with people with diabetes (telephone, email, and video consultations). Clinical diabetes services have been significantly disrupted, particularly in the areas of diabetes education, psychological support, and self-management support, with more modest disruptions in the areas of diabetes technology and medicine support.
13.	Fisher et al., 2020 [50]	United States	Cohort Study	The Taking Control of Your Diabetes (TCOYD) research registry	Around 40% reported that all of their diabetes-related appointments had been cancelled or postponed. About a third of respondents reported that laboratory tests had either been cancelled or postponed. 38% of respondents reported that one or more of their diabetes appointments had been switched to a virtual telehealth appointment. 45% of those who switched to telephone or video meetings reported lower satisfaction.
14.	Yeoh et al., 2020 [51]	Singapore	Cross-sectional	Primary care setting	During the pandemic and the lockdown, nearly all respondents were able to receive care safely from the clinics they attend (94%),and obtain their medications and diabetes equipment and supplies (97%) when needed. Most respondents were willing to explore tele-consultation options, and most of them indicated that they were able to reach their doctor through either phone, messaging, or email despite not attending clinic.

**Table 3:** Summary of study findings based on the derived themes.

No.	Author	Reduced Healthcare Utilization in Routine Healthcare Services	Surge of Telemedicine Usage	Delay in the Delivery of Healthcare Services
1.	Chen, Krupp, and Lo 2022 [38]	There were decreases in the proportion of patients who obtained HbA1c testing. There were decreases in both the proportion of patients with diabetes-related in-person office visits and the number of visits per patient. Overall utilization of non-emergent outpatient visits declined.	The proportion of patients with diabetes-related telehealth visits increased by 18%.	-
2.	Inglin et al., 2022 [39]	During the lockdown period, the number of diabetes-related contacts decreased significantly but quickly increased again to nearly the same level as in 2019. Overall, healthcare usage was lower in the pandemic year, with proportionally 9% fewer contacts per person and a proportionally 9% lower proportion of patients making any contact. Emergency visits went down significantly at the beginning of the lockdown period.	The proportion of remote consultations was similar in both years in the pre lockdown period (56.3–59.5%), but then increased to 88% during the 2020 lockdown.	-
3.	Maeda et al., 2022 [40]	-	-	There was a significant increase in delayed clinic visits during the pandemic, and women had significantly fewer clinic visits during the COVID-19 pandemic than men.
4.	Seidu et al., 2022 [41]	-	The most common consultation methods used to provide diabetes care during the pandemic were telephone consultation (92%), face-to-face consultation (80.2%), and video consultation (35%).	-
5.	Palanca et al., 2021 [42]	During full lockdown, about 50% of participants experienced a reduction in HbA1c testing, and the oldest participants were the most affected group.	-	-
6.	Al Harthi et al., 2021 [43]	-	Most patients received face-to-face consultation alone: 538 (57.4%), followed by combined face-to-face and telephone consultations: 304 (32.4%), and telephone consultation alone: 92 (10%). Most patients continued to receive diabetes care following the pandemic announcement by taking initiatives through phone consultation.	-
7.	Carr et al., 2021 [44]	In primary care, the rate of performing health checks was reduced by 76–88%, commonly among older people and low-income families.	-	-
8.	Mohseni et al., 2021 [45]	-	-	Outpatient and secondary care facilities have been pushed to limit or cancel their routine health service provision to mobilize healthcare providers to other high-pressure areas. Access to inpatient care is diminished for patients with other conditions.
9.	Yin et al., 2021 [46]	-	The COVID-19 pandemic has led to increased use of telemedicine.	
10.	Sciberras et al., 2020 [47]	-	-	Most outpatient services were temporarily halted during the pandemic, while those that continued their services were challenged due to staff reduction.
11.	Wicaksana et al., 2020 [48]	-	Emphasized the use of telehealth consultation for blood sugar monitoring, and telemedicine using mobile phones is useful for delivering diabetes education.	-
12.	Forde et al., 2020 [49]	-	Large increase in virtual contact with people with diabetes (telephone, email, and video consultations).	-
13.	Fisher et al., 2020 [50]	-	A large minority of the remaining participants reported that one or more of their diabetes appointments had been switched to a virtual telehealth appointment. 45% of those who switched to telephone or video meetings reported lower satisfaction.	Around 40% reported that all their diabetes-related appointments had been cancelled or postponed. About a third of the respondents reported that laboratory tests had either been cancelled or postponed.
14.	Yeoh et al., 2020 [51]	-	Most respondents were willing to explore tele-consultation options, and most of them indicated that they were able to reach their doctor through either phone, messaging, or email despite not attending clinic.	-

**Table 4:** Summary of reasons for reduced healthcare utilization in routine healthcare services.

No.	Author	Reduced Healthcare Utilization in Routine Healthcare Services	Reasons
1.	Chen, Krupp, and Lo 2022 [38]	There were decreases in the proportion of patients who obtained HbA1c testing. There were decreases in both the proportion of patients with diabetes-related in-person office visits and the number of visits per patient. Overall utilization of non-emergent outpatient visits declined.	1. COVID-19 pandemic.
2.	Inglin et al., 2022 [39]	During the lockdown period, the number of diabetes-related contacts decreased significantly but quickly increased again to nearly the same level as in 2019. Overall, healthcare usage was lower in the pandemic year, with proportionally 9% fewer contacts per person and a proportionally 9% lower proportion of patients making any contact. Emergency visits went down significantly at the beginning of the lockdown period.	1. The lockdown and restrictive measures affect the accessibility and organization of services drastically.
3.	Palanca et al., 2021 [42]	During full lockdown, about 50% of participants experienced a reduction in HbA1c testing, and the oldest participants were the most affected group.	1. Lockdown measures included restrictions on outdoor physical activity and social isolation. 2. Overwhelming work overload in primary care centers.
4.	Carr et al., 2021 [44]	In primary care, the rate of performing health checks was reduced by 76–88%, commonly among older people and low-income families.	1. COVID-19 restriction. 2. Reduction of clinical services.

## Data Availability

Not applicable.

## Supplementary Materials

The following supporting information can be downloaded at: https://www.mdpi.com/article/10.3390/ijerph20054577/s1. KIN 4400 Independent Research Study in Kinesiology|PRISMA 2020 Checklist; The Joanna Briggs Institute Critical Appraisal. References [33,38,39,40,41,42,43,44,45,46,47,48,49,50,51] are cited in the Supplementary Materials.
